# Measuring Coverage in MNCH: A Validation Study Linking Population Survey Derived Coverage to Maternal, Newborn, and Child Health Care Records in Rural China

**DOI:** 10.1371/journal.pone.0060762

**Published:** 2013-05-07

**Authors:** Li Liu, Mengying Li, Li Yang, Lirong Ju, Biqin Tan, Neff Walker, Jennifer Bryce, Harry Campbell, Robert E. Black, Yan Guo

**Affiliations:** 1 Johns Hopkins Bloomberg School of Public Health, Baltimore, Maryland, United States of America; 2 Guangxi Medical University, School of Public Health, Nanning, China; 3 Capital Medical University, School of Public Health, Beijing, China; 4 Gongcheng County Maternal and Child Health Hospital, Gongcheng County, China; 5 University of Edinburgh Medical School, Edinburgh, United Kingdom; 6 Peking University Health Science Center, School of Public Health, Beijing, China; Kings Colleage London, United Kingdom

## Abstract

**Background:**

Accurate data on coverage of key maternal, newborn, and child health (MNCH) interventions are crucial for monitoring progress toward the Millennium Development Goals 4 and 5. Coverage estimates are primarily obtained from routine population surveys through self-reporting, the validity of which is not well understood. We aimed to examine the validity of the coverage of selected MNCH interventions in Gongcheng County, China.

**Method and Findings:**

We conducted a validation study by comparing women’s self-reported coverage of MNCH interventions relating to antenatal and postnatal care, mode of delivery, and child vaccinations in a community survey with their paper- and electronic-based health care records, treating the health care records as the reference standard. Of 936 women recruited, 914 (97.6%) completed the survey. Results show that self-reported coverage of these interventions had moderate to high sensitivity (0.57 [95% confidence interval (CI): 0.50–0.63] to 0.99 [95% CI: 0.98–1.00]) and low to high specificity (0 to 0.83 [95% CI: 0.80–0.86]). Despite varying overall validity, with the area under the receiver operating characteristic curve (AUC) ranging between 0.49 [95% CI: 0.39–0.57] and 0.90 [95% CI: 0.88–0.92], bias in the coverage estimates at the population level was small to moderate, with the test to actual positive (TAP) ratio ranging between 0.8 and 1.5 for 24 of the 28 indicators examined. Our ability to accurately estimate validity was affected by several caveats associated with the reference standard. Caution should be exercised when generalizing the results to other settings.

**Conclusions:**

The overall validity of self-reported coverage was moderate across selected MNCH indicators. However, at the population level, self-reported coverage appears to have small to moderate degree of bias. Accuracy of the coverage was particularly high for indicators with high recorded coverage or low recorded coverage but high specificity. The study provides insights into the accuracy of self-reports based on a population survey in low- and middle-income countries. Similar studies applying an improved reference standard are warranted in the future.

*This paper is part of the* PLOS Medicine *“Measuring Coverage in MNCH” Collection*

## Introduction

Accurate data on coverage of key maternal, newborn, and child health (MNCH) interventions are crucial for monitoring progress toward the Millennium Development Goals 4 and 5 and ending preventable child deaths in a generation [1], [2]. Recognizing its significance, the Child Health Epidemiology Reference Group (CHERG) for WHO and UNICEF has made it a priority to improve coverage measurement for proven MNCH interventions. This paper is part of the *PLOS Medicine* “Measuring Coverage in MNCH” collection organized by CHERG for this purpose. Coverage estimates are generally obtained from routine population-based household surveys, such as the Demographic and Health Surveys (DHS) and the Multiple Indicator Cluster Surveys (MICS), primarily through self-reporting [3]. However, little is known about the validity of self-reported coverage derived from these population-based surveys.

Previous validation studies comparing health care records with respondents’ self-reports were mostly conducted in facility-based settings in high-income countries and found varying and often moderate validity across MNCH indicators studied. For example, an Australian study comparing medical records with women’s reports of delivery interventions found that women’s self-reports and medical records were both subject to errors [4]. In another recent population-based survey conducted in the UK, self-reported delivery mode was found to be highly reliable [5]. Two studies done in American hospitals showed generally unsatisfactory validity of self-reported medical interventions in the pregnancy, delivery, and postnatal periods [6], [7], while one study among mothers of children with cancer in Canada and the US reported moderate to high validity for similar indicators [8]. According to one other US study, parents’ reports of children’s immunizations were of unsatisfactory validity, mainly due to poor initial encoding of the events [9].

Results from high-income countries may have limited generalizability when applied to the low- and middle-income countries (LMICs) due to different levels of coverage, intensities of service provision counseling, and degrees of recall bias that may be associated with the education level of respondents [10], [11]. However, similar studies are sparse in the LMIC setting; most of them have focused on obstetrical complications rather than routine interventions [12]–[14]. To our knowledge, the current study and other validation studies in this collection are the only ones that aim to evaluate the accuracy of coverage of MNCH interventions in LMICs [15]–[18].

Most of the validation studies reviewed are facility-based, and therefore subject to selection biases, because the study sample is often not representative of the general population. The fact that such a facility-based study design is widely adopted is perhaps because validation studies based on population surveys are more methodologically challenging. This is particularly true in LMICs, because health-care recordkeeping systems are rarely complete or of adequate quality to be used as the reference standard. In this study, we sought to examine the validity of self-reported coverage of selected MNCH interventions in a relatively less developed rural area in China, a setting selected to increase the extent to which the study results would be generalizable to other LMICs. In addition, we attempted to minimize selection bias associated with facility-based validation studies by collecting the study sample through a population-based survey.

## Methods

### Study Site

We conducted the validation study in Gongcheng County, which is located in Guangxi Province in southwestern China. The county contains nine townships and 125 villages [19], with a total population of 285,058 based on a 2010 census, of which 59% were of Yao and 39% were of Han ethnicity [20]. Among the population aged 15 and above, 1.2% were illiterate. The majority of the population were fruit farmers and engage in citrus and persimmon production. In 2006, the county GDP per capita was reported to be around $1,500 [19].

In 2006–2010, the under-five mortality rate in Gongcheng County decreased from 15.2 to 8.1 per 1,000 live births, and the infant mortality rate declined from 13.4 to 6.8 per 1,000 live births [21]. During the same period, the maternal mortality ratio was on average 31.8 per 100,000 live births [21]. Most MNCH services are provided in county- and township-level hospitals and village clinics [22]. Coverage of antenatal care and institutional delivery is close to universal in the past five years [21]. Information on MNCH services is routinely recorded by service providers in a number of booklets, including, for example, the antenatal care booklet, the maternal and child health booklet, the child care booklet, and the vaccination booklet. The antenatal and child care booklets have been in use since 2003. These booklets are usually kept by women. In January 2007, an electronic MNCH information system was launched as part of the Guangxi Province MNCH information system. The system digitized key information collected from the booklets.

### Data Collection

Women aged 18 to 49 years who lived in Gongcheng County during the fieldwork and had delivered at least one live birth in the county in the five years preceding the survey (i.e., between 01 November 2006 and 01 November 2011) were eligible to participate in the study. Participants were selected via multi-stage stratified sampling with a target of interviewing mothers of 1,000 live births. The target sample size was determined based on the following consideration. The study was originally designed to also evaluate whether the validity of women’s self-reports was worse based on a five-year compared to a two-year recall period. The study sample size needed to be sufficient to distinguish ten percentage point differences in validity (e.g., sensitivity) when comparing the two recall periods. Ten percent is considered to be programmatically important. Since no prior information was available on the sensitivity of the measurement of any indicators studied, 50% sensitive was assumed for the five-year recall period to yield the most conservative sample size. Based on a 10% difference, 60% sensitivity was assumed for the two-year recall. Assuming constant fertility, the number of live births born in the past two years is two-fifths of those born in the past five years. To ensure that the sample size was conservative, continuation correction was applied to improve the approximation of binomial distribution to the normal distribution [23]. With a significance level of 0.05 and 80% of power, sample size calculation for two-sample comparison of proportions using Stata 10 produced a total sample size of 714 live births in the past five years and 286 live births in the past two years [24]. We assumed that coverage of 20% of the study sample cannot be validated due to the lack of the reference standard. Taking into account a 10% non-response rate, a total of 992 or approximately 1,000 live births were needed. Information on 900 live births was anticipated to be actually collected.

Study women were selected via a multi-stage stratified sampling design. In the first stage, the nine townships were divided into three strata based on the population size as a proxy of the level of economic development, and one township was selected in each stratum. In the second stage, villages were divided into four groups according to their geographic location (east, west, north, and south), and one village was sampled from each geographic group. In the third stage, participants were recruited for interview by the village doctors based on availability by going through the vaccination roster, which is considered to have enlisted all children under five years of age in the villages. Recruitment stopped once the desired sample size in each township was reached. During the recruitment process, women were also asked to bring their MNCH booklets to the community interview for abstraction.

The study period overlapped with the persimmon harvesting season, which made it difficult to recruit participants based on our original plan. However, the fieldwork happened to fall on child immunization days in two of the three sampled townships, during which young children were brought to the township hospitals for immunization and well-baby checkup. A fraction of the study sample was recruited on the immunization days at the end. As a result, children younger than two years were over-represented in the study sample.

Community-based face-to-face interviews were conducted in village centers with reasonable privacy after obtaining written informed consent. The survey instrument was adapted from the DHS and MICS questionnaires to suit the local context [25]. It was used to solicit information on coverage of selected MNCH interventions, including those routinely collected in the DHS or MICS or of local relevance. Some additional modifications were made in the wording of a number of questions, including those on child vaccination, in an effort to reduce the length of the questionnaire. The exact wording of the questions used in the survey in comparison with those used in the DHS and MICS questionnaires is provided in Table S1. The questionnaires were designed in English, translated into Chinese, and verified through back-translation. One questionnaire was administered to each eligible woman to collect information on household characteristics and her socio-demographic background. A second questionnaire was administered for each eligible live birth to collect information on services received during the antenatal, delivery, newborn, postnatal, and child care periods.

We abstracted relevant records from available booklets after completion of the interview using a structured template. We also extracted relevant records from the electronic system for all women residing in Gongcheng who delivered locally between the initiation of the electronic system (01 January 2007) and 01 October 2011. Because study women may not have all booklets available for abstraction, the electronic system was not in operation for the first few months of the study reference period, and a small set of indicators were only available in the booklets, we combined records from booklet abstraction and the electronic systems. The study reference standard was created by giving preference to the electronic system and is referred to as Gongcheng MNCH Information System (GMNCHIS). Only indicators for which information was available from GMNCHIS were included in the validation. A complete list of validated indicators can be found in Table S1.

### Data Analyses

We cleaned and matched data collected through the community survey and those abstracted from the GMNCHIS. A number of databases were exported from the electronic system, including pregnant women’s general information, early antenatal care, other antenatal care, high-risk pregnancy, antenatal screening tests, delivery, postnatal, and child care databases. When processing the exported data, record of results of a test or examination was treated as the evidence of the receipt of the test or examination, and the lack of such a record was treated as evidence of not receiving the service. Records in different databases were matched using the maternal and child health identification unique for each pregnancy. Record of receipt of a service in any of the databases was considered evidence of the receipt of the service.

To identify potentially duplicated records for the same pregnancy in the electronic system, we first identified records for the same woman, defined as records with either the same national identification or the same name and village, and less than four years of differences in reported age. We then identified the records for the same pregnancy, defined as records of the same woman who had first antenatal visits fewer than 30 days apart, or last menstrual periods fewer than 30 days apart, or delivery records fewer than 150 days apart so that a pregnancy loss before another pregnancy was not identified as the same pregnancy. Lastly, we collapsed all the duplicated records for the same pregnancy. A total of 15,189 unique women with 16,049 unique pregnancies for the whole county were identified in the electronic system.

To match women’s recall in the community-based survey with those collected through the GMNCHIS, we first matched survey data with those exported from the electronic system using the following combinations of information: (1) maternal and child health identification and women’s names, or (2) first 14 digits of the national identification which includes an area code, date of birth, women’s names, and children’s date of birth, or (3) women’s names, village names, and children’s date of birth. Then we used data abstracted from the booklets as the reference standard for those women and indicators which were not matched using data exported from the electronic system. Our unit of analysis was live birth.

We grouped the results into four categories for presentation, including antenatal care, delivery care, postnatal care, and child vaccination services. Antenatal care includes routine antenatal care, blood screening for sexually transmitted diseases, and blood screening for congenital abnormalities. During the routine antenatal care, the first ultrasound scan was done on the first antenatal visit, usually around 10–14 weeks. It provided information on gestational age and examined fetal conditions, including measuring nuchal translucency to screen for Down’s syndrome. Normally, a few antenatal tests are done during each antenatal visit, including weight/height/blood pressure measurements, urine test, and fetal heart monitoring. For these repeated tests, we only measured whether they were received at least one time. Pregnant women are screened for thalassemia by a combination of mean cell volume count, erythrocyte osmotic fragility test, and hemoglobin electrophoresis [26], [27].

We calculated sensitivity and specificity of the self-reported coverage. We graphed the overall validity in a receiver operating characteristic (ROC) plot with true positive rate, or sensitivity, plotted against false positive rate, or one minus specificity. We also quantified the overall validity by the area under the ROC curve (AUC) [28]. The uncertainty associated with validity, as represented by the 95% confidence interval (CI), was estimated assuming a binomial distribution.

Population-level accuracy of the coverage estimates was also examined, which is measured by the test to actual positive (TAP) ratio or the reported over-recorded coverage [29]. It can be demonstrated mathematically that the TAP ratio is determined by the validity of self-reported coverage in combination with the actual (or recorded) coverage [29]. If the recorded coverage is high, the TAP ratio approximately equals sensitivity and is independent of specificity [29]. A combination of low recorded coverage and low to moderate specificity results in a high TAP ratio [29], [30]. We also investigated and discussed the complex mathematical relationship observed between the TAP ratio, validity, and recorded coverage.

For the purpose of describing the study results, we categorized coverage, sensitivity, and specificity as low, moderate, and high, applying two cut-off points at 0.33 and 0.66. We also considered the overall validity high if the AUC was at or above 0.67 and moderate and low otherwise. We qualitatively defined the population-level bias based on the TAP ratio as small (0.8<TAP ratio<1.2), moderate (0.5<TAP ratio<1.5), and large (TAP ratio<0.5 or TAP ratio>1.5). We also conducted two sensitivity analyses to treat information solely abstracted from the booklets as the reference standard and to limit the study sample to women who gave birth in the past two years.

### Ethical Review

The study protocol was approved by the Institutional Review Boards of the Johns Hopkins Bloomberg School of Public Health and Peking University.

## Results

### Characteristics of the Study Sample

Nine hundred and thirty-six women were recruited between 10 and 22 November 2011. Among them, 914 agreed to participate in the survey and delivered 994 eligible live births. Interviews on 961 eligible live births were completed, among whom mothers of 431, 115, 343, and 793 live births brought the antenatal, maternal and child health, child care, and vaccination booklets to the community survey, respectively. Seven hundred and twelve live births were matched using electronic information. Another 196 live births were matched using information from at least one booklet, yielding a total of 908 matched live births. The remaining 53 live births did not have any matched indicators and could not be validated.

The socio-demographic characteristics of the surveyed live births by matching status are presented in Table 1. Overall, mothers of almost 60% live births were aged between 25 and 34 years, and of more than half had secondary or higher education. Similar to Gongcheng’s general population, mothers of 59% of live births were of Yao ethnicity. The majority (84%) of the live births sampled was the only one born in the past five years and 42% were under one year old. More than half of the live births lived in households with an annual income per capita ranging between 1,000 and 5,000 Yuan, or 158 and 791 dollars.

**Table 1:** Socio-demographic characteristics of surveyed live births by matching status.

Category	Total		Matched		Unmatched		p-Value^a^
	No.	%	No.	%	No.	%	
**Mother’s age, years**							0.164
24 and below	162	16.9%	159	17.5%	3	5.7%	
25–29	317	33.1%	299	33.0%	18	34.0%	
30–34	251	26.2%	232	25.6%	19	35.8%	
35–39	161	16.8%	151	16.7%	10	18.9%	
40 and above	68	7.1%	65	7.2%	3	5.7%	
Total^b^	959	100.0%	906	100.0%	53	100.0%	
**Mother’s education**							0.139
Primary	336	35.0%	311	34.3%	25	47.2%	
Secondary	510	53.1%	486	53.5%	24	45.3%	
Tertiary	115	12.0%	111	12.2%	4	7.5%	
Total	961	100.0%	908	100.0%	53	100.0%	
**Mother’s ethnic group**							0.045
Yao	569	59.2%	546	60.1%	23	43.4%	
Han	340	35.4%	313	34.5%	27	50.9%	
Other	52	5.4%	49	5.4%	3	5.7%	
Total	961	100.0%	908	100.0%	53	100.0%	
**Number of births in the last 5 years**							<0.001
1	806	84.2%	780	86.3%	26	49.1%	
2	147	15.4%	120	13.3%	27	50.9%	
3	4	0.4%	4	0.4%	0	0.0%	
Total^b^	957	100.0%	904	100.0%	53	100.0%	
**Children’s age, months**							<0.001
Below 12	404	42.0%	401	44.2%	3	5.7%	
12–23	192	20.0%	188	20.7%	4	7.5%	
24–35	138	14.4%	128	14.1%	10	18.9%	
36–47	112	11.7%	105	11.6%	7	13.2%	
48–59	115	12.0%	86	9.5%	29	54.7%	
Total	961	100.0%	908	100.0%	53	100.0%	
**Annual household income per capita, Yuan**							0.373
Below 1,000	104	14.4%	99	14.6%	5	10.6%	
1,000–1,999	157	21.7%	143	21.1%	14	29.8%	
2,000–4,999	253	34.9%	235	34.7%	18	38.3%	
5,000–9,999	141	19.5%	136	20.1%	5	10.6%	
10,000 and above	69	9.5%	64	9.5%	5	10.6%	
Total^b^	724	100.0%	677	100.0%	47	100.0%	

a Based on the Chi-square test comparing matched and unmatched samples.

b Live births born to mothers who answered “don’t know” to or refused to answer the corresponding questions are not presented.

Mother’s age, education, and household annual income per capita were not significantly different between the matched and unmatched live births. However, the matched live births were more likely to have been born to mothers of Yao ethnicity, to be the only live birth in the last five years, and to be younger than 24 months.

### Validity of the MNCH Indicators

Reported coverage derived from the community survey, recorded coverage derived from the GMNCHIS and TAP ratio are reported in Table 2. Sensitivity, specificity, AUC, and their corresponding 95% CI are presented in Table 3. The reported coverage of the routine antenatal care indicators was high (>81%). Their recorded coverage was also high, with the exception of the first antenatal visit before 12 weeks of gestational age. Self-reported coverage of routine antenatal interventions had sensitivity close to 0.90 and specificity below 0.25. Recorded coverage of the antenatal HIV and hepatitis B antibody (HBsAb) tests was similar, although their reported coverage differed greatly. Self-reported coverage of the HIV test had moderate sensitivity (0.59 [95% CI: 0.54–0.65]) and specificity (0.53 [95% CI: 0.46–0.60]), whereas that of the HBsAb test had high sensitivity (0.89 [95% CI: 0.86–0.92]) and low specificity (0.18 [95% CI: 0.13–0.22]). Among the screening tests for Down’s syndrome, neural tube defects, and thalassemia, despite similar levels of reported coverage of 54%–68%, the recorded coverage varied, ranging between 18% and 52%. Self-reported coverage of each of these screening tests had sensitivities of 0.75–0.87 and specificities of 0.46–0.52.

**Table 2:** Coverage of selected MNCH indicators.

Indicators	Number of Observations			Coverage		
	Missing^a^	Indeter-minate^b^	Validated	GMNCHIS	CommunitySurvey	TAP Ratio
**Routine antenatal care**						
First antenatal care <12 weeks of gestational age	194	86	675	0.31	0.81	2.66
At least four antenatal visits	176	246	539	0.70	0.91	1.30
Weight measurement	165	14	782	1.00	0.98	0.98
Height measurement	182	10	769	0.96	0.89	0.92
Blood pressure measurement	165	6	790	0.99	0.98	0.99
Hemoglobin test	132	53	776	0.85	0.92	1.09
Urine test	166	8	787	0.93	0.99	1.06
Fetal heart rate monitoring	175	6	780	0.99	0.99	1.00
Ultrasound exam	189	4	768	0.86	0.99	1.15
**Screening for STD**						
HIV test	256	152	553	0.60	0.54	0.91
HBsAb test	175	86	700	0.59	0.86	1.46
**Screening for congenital abnormalities**						
Down’s syndrome screening	244	206	511	0.18	0.59	3.21
Neural tube defect screening	200	228	533	0.20	0.54	2.73
Thalassemia screening	769	41	151	0.52	0.68	1.32
**Delivery care**						
Cesarean section	184	0	777	0.24	0.36	1.47
**Postnatal care**						
At least 1 postnatal visit	231	3	727	0.29	0.37	1.25
Blood pressure	810	3	148	0.83	0.87	1.05
Temperature	854	3	104	0.78	0.87	1.11
Breast exam	810	2	149	0.83	0.66	0.80
Uterus exam	810	5	146	0.83	0.77	0.93
Lochia exam	810	2	149	0.83	0.77	0.93
Perineum exam	811	3	147	0.84	0.81	0.97
Family planning advice	821	1	139	0.68	0.75	1.11
**Child vaccination**						
BCG vaccine	240	151	570	0.91	0.94	1.04
Polio vaccine	258	14	689	1.00	0.86	0.86
HBV vaccine	256	31	674	0.92	0.98	1.07
DPT vaccine	230	273	458	0.75	0.74	0.99
Measles vaccine	253	206	502	0.35	0.70	2.00

a Missing indicates live births who were not matched.

b Indeterminate indicates live births whose mothers answered “Don’t know” or refused to give an answer to the corresponding survey questions.

BCG, Bacillus Calmette-Guérin; DPT, diphtheria-pertussis-tetanus; HBV, hepatitis B virus.

**Table 3:** Validity of selected MNCH indicators.

Indicators	Sensitivity		Specificity		AUC		
	Est.	95% CI	Est.	95% CI	Est.	95% CI	
**Routine antenatal care**							
First antenatal visit <12 weeks of gestational age	0.90	(0.86, 0.94)	0.22	(0.19, 0.26)	0.56		(0.54, 0.59)
At least 4 antenatal visits	0.98	(0.96, 0.99)	0.25	(0.19, 0.32)	0.62		(0.58, 0.65)
Weight measurement	0.98	(0.97, 0.99)	0.00	(0.00, 0.00)	0.49		–
Height measurement	0.89	(0.87, 0.91)	0.23	(0.08, 0.37)	0.56		(0.48, 0.63)
Blood pressure measurement	0.98	(0.97, 0.99)	0.00	(0.00, 0.00)	0.49		(0.49, 0.50)
Hemoglobin test	0.93	(0.91, 0.95)	0.14	(0.08, 0.21)	0.54		(0.50, 0.57)
Urine test	0.99	(0.98, 1.00)	0.02	(0.00, 0.05)	0.50		(0.48, 0.52)
Fetal heart rate monitoring	0.99	(0.98, 1.00)	0.10	(0.00, 0.29)	0.54		(0.45, 0.64)
Ultrasound exam	0.99	(0.98, 1.00)	0.01	(0.00, 0.03)	0.50		(0.49, 0.51)
**Screening for STD**							
HIV test	0.59	(0.54, 0.65)	0.53	(0.46, 0.60)	0.54	(0.51, 0.56)	
HBsAb test	0.89	(0.86, 0.92)	0.18	(0.13, 0.22)	0.56	(0.52, 0.60)	
**Screening for congenital abnormalities**							
Down's syndrome screening	0.81	(0.73, 0.88)	0.46	(0.41, 0.5.0)	0.63	(0.59, 0.68)	
Neural tube defect screening	0.75	(0.67, 0.84)	0.51	(0.46, 0.56)	0.63	(0.58, 0.68)	
Thalassemia screening	0.87	(0.80, 0.95)	0.52	(0.41, 0.64)	0.70	(0.63, 0.76)	
**Delivery care**							
Cesarean section	0.96	(0.93, 0.99)	0.83	(0.80, 0.86)	0.90	(0.88, 0.92)	
**Postnatal care**							
At least one postnatal visit	0.57	(0.50, 0.63)	0.72	(0.68, 0.76)	0.64	(0.60, 0.68)	
Blood pressure	0.90	(0.85, 0.95)	0.28	(0.10, 0.46)	0.59	(0.50, 0.68)	
Temperature	0.93	(0.87, 0.98)	0.35	(0.15, 0.54)	0.64	(0.53, 0.74)	
Breast exam	0.66	(0.57, 0.74)	0.35	(0.16, 0.53)	0.50	(0.40, 0.60)	
Uterus exam	0.76	(0.68, 0.84)	0.20	(0.04, 0.36)	0.48	(0.39, 0.57)	
Lochia exam	0.78	(0.71, 0.85)	0.28	(0.10, 0.46)	0.53	(0.43, 0.63)	
Perineum exam	0.81	(0.74, 0.88)	0.21	(0.05, 0.37)	0.51	(0.42, 0.60)	
Family planning advice	0.78	(0.69, 0.86)	0.31	(0.18, 0.45)	0.54	(0.46, 0.62)	
**Child vaccination**							
BCG vaccine	0.96	(0.94, 0.97)	0.24	(0.13, 0.35)	0.60	(0.54, 0.66)	
Polio vaccine	0.86	(0.84, 0.89)	–	–	–	–	
HBV vaccine	0.98	(0.97, 0.99)	0.02	(0.00, 0.05)	0.50	(0.48, 0.52)	
DPT vaccine	0.89	(0.86, 0.92)	0.70	(0.61, 0.78)	0.80	(0.75, 0.84)	
Measles vaccine	0.95	(0.92, 0.98)	0.44	(0.38, 0.49)	0.69	(0.66, 0.72)	

BCG, Bacillus Calmette-Guérin; DPT, diphtheria-pertussis-tetanus; HBV, hepatitis B virus.

Coverage of cesarean section was reported to be 36%, compared to the recorded 24%. It had high sensitivity (0.96 [95% CI: 0.93–0.99]) and specificity (0.83 [95% CI: 0.80–0.86]). Among the postnatal care indicators, coverage of occurrence of at least one postnatal visit was reported to be higher than the recorded value, with a moderate sensitivity (0.57 [95% CI: 0.50–0.63]) and a high specificity (0.72 [95% CI: 0.68–0.76]). The rest of the postnatal indicators had high reported and recorded coverage, with moderate to high sensitivity (0.66–0.93) and low to moderate specificity (0.21–0.35). Reported and recorded coverage of vaccination was consistently high, with the exception of measles vaccine. Self-reported coverage of vaccination also had high sensitivity (>0.86) and a wide range of specificity (0.02–0.70).

**Figure 1:**
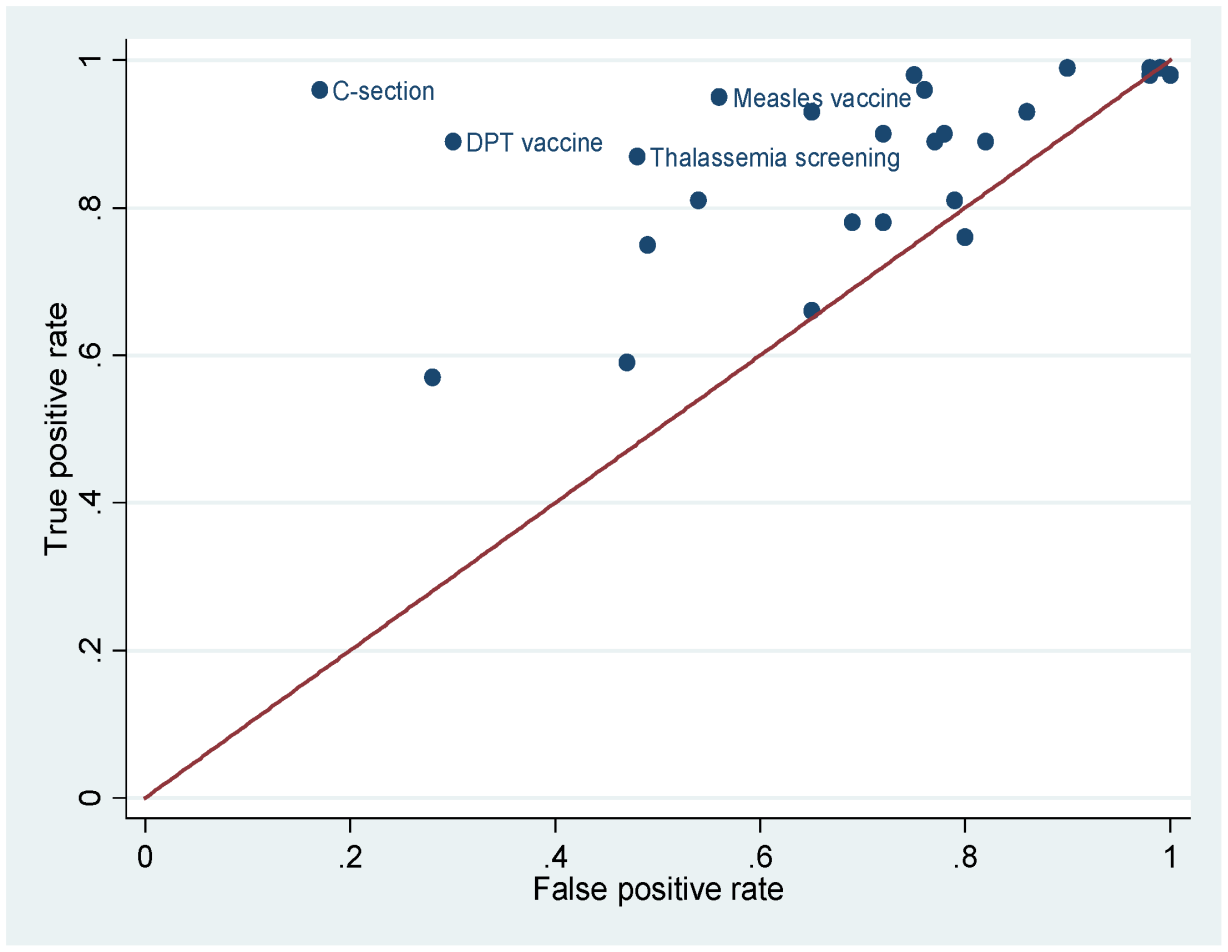
ROC plot of validated indicators. The red line indicates the diagonal.

The AUC estimates reported in Table 3 and the ROC plot shown in Figure 1 demonstrate the overall validity of self-reported coverage by indicator. Self-reported coverage of cesarean section had the highest overall validity when compared to the reference standard, with the AUC being 0.90 [95% CI: 0.88–0.92]. Diphtheria-pertussis-tetanus (DPT) vaccine ranked the second, with the AUC being 0.80 [95% CI: 0.75–0.84]. Self-reported coverage of thalassemia screening and measles vaccine also had high overall validity (AUC>0.69). The remaining indicators had either moderate or low overall validity, with the AUC of a number of them not significantly different from 0.5, indicating validity equivalent to a random guess.

**Figure 2:**
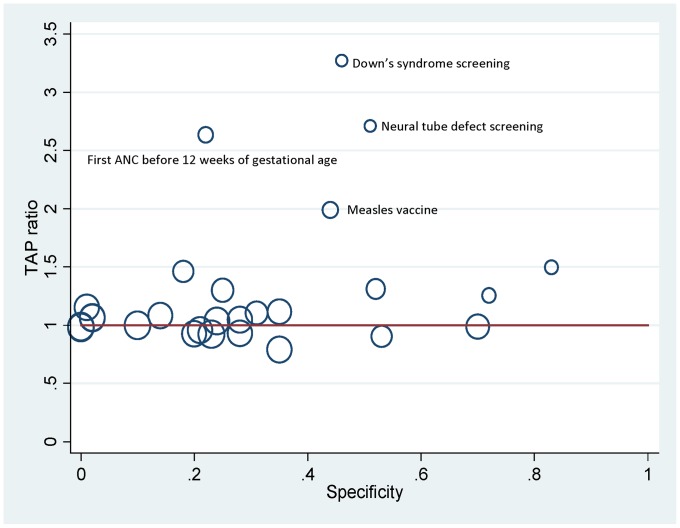
TAP ratio by specificity of self-reported coverage of selected MNCH indicators. The size of the circle represents the recorded coverage in the GMNCHIS.

Despite varying overall validity, the TAP ratios ranged between 0.8 and 1.5 for self-reported coverage of 24 of the 28 indicators examined, suggesting mostly small to moderate degree of bias at the population level (Figure 2). However, it was particularly large for four indicators, including measles vaccine (TAP ratio = 2.0), first antenatal visit before 12 weeks of gestational age (TAP ratio = 2.7), screening for neural tube defect (TAP ratio = 2.7), and screening for Down’s syndrome (TAP ratio = 3.2). Both sensitivity analyses for using the booklets as the reference standard and limiting the sample to women who gave birth in the last two years gave quantitatively similar results (not shown).

## Discussion

To our knowledge, this is the first study to validate self-reported coverage of a range of MNCH indicators by systematically comparing women’s self-reports solicited from a population-based survey of MNCH care records in a LMIC. We found that across the indicators examined, self-reported coverage had moderate to high sensitivity and low to moderate specificity. The overall validity is high for the self-reported coverage of a few indicators including cesarean section, diphtheria-pertussis-tetanus vaccine, measles vaccine and screening for thalassemia, yet moderate to low for the remaining indicators. The finding of moderate levels of overall validity is not unexpected, as similar results have been reported in previous studies in high-income countries [6], [9].

The variation in validity across indicators seems to suggest that the more distinctive the experiences women had while receiving certain interventions, the better was the validity of the self-reported coverage. The positive association between event distinctiveness and recall accuracy is supported by the psychology literature [31]. The variation could also be the result of the social desirability bias associated with self-reports. That is, when women perceived that it was socially desirable to receive a certain service, they were more likely to report receipt of the service regardless of whether they had actually received it or not. An example illustrating the potential social desirability bias can be drawn from the coverage and validity of the HIV and HBsAb tests. The two tests had similar levels of recorded coverage, yet widely different levels of reported coverage. We hypothesize that women may be less willing to report receipt of an HIV test than an HBsAb test, as the former is less socially desirable.

Despite varying validity, self-reported coverage of the majority of the examined indicators had only a small to moderate degree of population-level bias. At least two reasons can perhaps explain this. The first reason is based on the mathematical relationship between bias, validity, and the recorded coverage. It can be demonstrated that when recorded coverage is high, the TAP ratio approximately equals sensitivity and is independent of specificity [29]. Because a large proportion of the indicators had high recorded coverage and high sensitivity, their TAP ratio did not deviate greatly from 1. Of note, although high coverage may have limited our power to accurately estimate specificity, the accuracy of coverage is not much affected, as specificity is almost irrelevant to population-level bias when coverage is high.

The second reason is better recall and recognition of certain interventions due to better community knowledge associated with high coverage. We observed that the higher the recorded coverage is, the higher the sensitivity is, and the correlation is marginally significant (p = 0.06). This should not be the case under normal circumstances, as sensitivity and specificity are in theory intrinsic to the estimation of self-reported coverage, and are usually independent of the actual coverage [32]. As a result, high coverage is also likely associated with high sensitivity.

On the other hand, the self-reported coverage of a few indicators had large bias, including screening for Down’s syndrome and neural tube defects, first antenatal visit before 12 weeks of gestational age, and measles vaccine. This is likely due to a similar mathematical relationship–a combination of low recorded coverage and low to moderate specificity results in a high TAP ratio [29], [30]. In fact, for the current recorded coverage and sensitivity of screening for Down’s syndrome, for example, specificity needs to be as high as 0.96, compared to the current 0.46, to yield a TAP ratio of 1 [29]. For a combination of low prevalence and low specificity, coverage derived from self-reports in population-based surveys always overestimates the actual coverage. The degree of overestimation increases with the decrease of the actual coverage and specificity.

In summary, despite moderate and varying validity, the population-level bias in coverage estimates was mostly small to moderate in this study, particularly for indicators with high recorded coverage or low recorded coverage but high specificity. Of note, although the bias may not be large at the population level, the degree of misclassification at the individual level could still be large due to unsatisfactory validity of some indicators.

Our study is subject to a number of limitations. First, our reference standard has some caveats. The fact that the self-reported coverage of a number of indicators had low or lower than expected specificity, including that of cesarean section which would normally have closer to 100% specificity, suggests that the quality of the GMNCHIS is perhaps not optimal. For a distinctive event like cesarean section, self-reporting might even be more reliable. MNCH services received outside the study county may not be recorded in the GMNCHIS, although one had to deliver in Gongcheng to be included in the study. Anecdotal evidence also suggests that the completeness of the electronic system has improved over time since its initiation in 2007. This was further supported by the finding that live births were significantly less likely to be matched using the electronic system in 2007–2009 compared to more recent years (p<0.001). However, our sensitivity analysis shows that the validity is the same between women who gave birth in the past five years and those in the past two years. This suggests that although the completeness may be lower in 2007–2009, among women recorded in the electronic system, the validity of their self-reports in the past five years is similar to that in the past two years. If however, self-reports of women that were not captured by the electronic system in its early stage had very different validity from those captured later, the limited completeness may introduce bias to our findings, although the direction is difficult to determine.

Second, our study sample may not necessarily be representative of the whole county. The study participants were drawn from village vaccination rosters, which may have missed children born outside of the national family planning policy. However, this bias is likely to be small as Gongcheng is a minority-concentrated area and such women could usually have up to two children. In addition, within the primary sampling units, study participants were recruited based on availability by going through the vaccination rosters until the desired sample size was reached. This process may not be completely random, which could introduce selection bias, although we do not have reason to believe that the bias is systematic. The fact that our matched and unmatched study sample differed in mothers’ ethnicity, number of live births in the past five years, and children’s age may also introduce bias if these characteristics are associated with recall accuracy. However, the lack of representativeness does not affect the validation results, but may limit the study generalizability.

Third, there are other factors that may limit our generalizability. The study was conducted in a setting where coverage of selected MNCH indicators was in general higher than that in countries where DHS and MICS surveys are normally conducted. In addition, we conducted this study in an area that is relatively more developed than those where most DHS and MICS surveys are usually conducted. If socio-economic development factors, such as education, are associated with validity as found previously [10], [11], the study results could not be directly applied to other settings with different development levels. Despite these limitations, our study results could be subject to fewer selection biases and be more generalizable than other facility-based studies in this Collection, although they would have a higher-quality reference standard based on direct observation [15]–[18].

Factors associated with the design and implementation of the survey may also affect the external validity of the study. We interviewed women in a central location in the community rather than in their households, which may affect validity of certain indicators. However, we speculate that this influence is likely to be small for the indicators studied, most of which are not sensitive at all in this context. Validity or reliability of questions included in the survey instruments could also affect the study’s internal and external validity. For instance, we failed to include the age limit of the measles vaccine in the questionnaire, which is 8 months or older in China [33]. As a result, the coverage of measles vaccine had high false positive rate and large bias. It is illustrated by the fact that children older than 8 months only constituted 60% of the matched live births, whereas the measles vaccine coverage rate was reported to be 70%, which is unlikely to be true.

In conclusion, more population-based validation studies are warranted with an improved reference standard and survey instruments. Future research should further examine the generalizability of observed validity to other LMIC settings. Nevertheless, the current study contributes to our understanding of validity of self-reported coverage of a range of MNCH interventions. It provides insights into the population-level accuracy of self-report based on a population survey in the LMICs.

## Supporting Information

Table S1 List of the 28 matched indicators and the corresponding questions used in the community questionnaire, in comparison with those used in the DHS and MICS.
(DOCX)

## Abbreviations

AUC: area under the receiver operating characteristic curve
CHERG: Child Health Epidemiology Reference Group
CI: confidence interval
DHS: Demographic and Health Survey/s
GMNCHIS: Gongcheng MNCH Information System
HBsAb: hepatitis B antibody
LMIC: low- and middle-income country
MICS: Multiple Indicator Cluster Surveys
MNCH: maternal, newborn, and child health
ROC: receiver operating characteristic
TAP: test to actual positive
