# Supplementary material for: Measuring Coverage in MNCH: A Validation Study Linking Population Survey Derived Coverage to Maternal, Newborn, and Child Health Care Records in Rural China
Source: PLoS One. 2013 May 7;8(5):e60762. doi: 10.1371/journal.pone.0060762 (PMC3646215; doi:10.1371/journal.pone.0060762)
Supplement: Table S1 — List of the 28 matched indicators and the corresponding questions used in the community questionnaire, in comparison with those used in the DHS and MICS. (DOCX) [file pone.0060762.s001.docx]

Table S1. List of the 28 matched indicators and the corresponding questions used in the community questionnaire, in comparison with those used in the DHS and MICS

| **Indicators** | | **China MNCH service validity** | **DHS (round 5 and 6)** | **MICS (round 3)** |
| --- | --- | --- | --- | --- |
| **ANC** | |  |  |  |
|  | **First ANC before 12 weeks of gestational age** | How many months pregnant were you when you first received antenatal care for this pregnancy? | How many months pregnant were you when you first received antenatal care for this pregnancy? |  |
|  | **At least 4 ANC visits** | How many times did you receive antenatal care when you were pregnant with [NAME]? | How many times did you receive antenatal care during this pregnancy? |  |
|  | **Weight measurement** | As part of your antenatal care during your pregnancy with [NAME], were any of the following done at least once:  Were you weighed? | As part of your antenatal care during this pregnancy, were any of the following done at least once:   Were you weighed? (only in round 5) | As part of your antenatal care during this pregnancy, were any of the following done at least once:  Were you weighed? |
|  | **Height measurement** | Were you measured for height? |  |  |
|  | **Blood pressure measurement** | Was your blood pressure measured? | Was your blood pressure measured? | Was your blood pressure measured? |
|  | **Blood test** | Did you give a blood sample? | Did you give a blood sample? | Did you give a blood sample? |
|  | **Urine test** | Did you give a urine sample? | Did you give a urine sample? | Did you give a urine sample? |
|  | **Fetal heart rate monitoring** | As part of your antenatal care during your pregnancy with [NAME], were any of the following done at least once:  Did the provider hear the heart of the fetus? |  |  |
|  | **Ultra-sound exam** | Did the provider use ultrasound B to examine the fetus? |  |  |
| **STD screening** | |  |  |  |
|  | **HIV test** | Did you give a blood sample to test for any of the following:   HIV? | Were you offered a test for the AIDS virus as part of your antenatal care? | I don’t want to know the results, but were you tested for hiv/aids as part of your antenatal care? |
|  | **Hbsag test** | Hepatitis B? |  |  |
| **Congenital malformation screening** | |  |  |  |
|  | **Down's Syndrome screening** | Did you give a blood sample to test for any of the following:   Down’s syndrome of the fetus |  |  |
|  | **Neural tube defect screening** | Neural tube defect of the fetus |  |  |
|  | **Thalassemia screening** | Thalassemia of the fetus? |  |  |
| **Delivery care** | |  |  |  |
|  | **Caesarean section** | Was [NAME] delivered by caesarean section? | Was [NAME] delivered by caesarean section? (round 5)  Was (NAME) delivered by caesarean, that is did they cut your belly open to take the baby out? (round 6) |  |
| **Postnatal care** | |  |  |  |
|  | **1+ postnatal visit** | Within 42 days after [NAME] was born, did you seek postnatal care for yourself? | After you were discharged, did any health care provider or traditional birth attendant check on your health? (round 5)  Did anyone check on your health after you left the facility? (round 6) |  |
|  | **Blood pressure** | Did the provider exam any of the following for you:   Blood pressure? |  |  |
|  | **Temperature** | Temperature? |  |  |
|  | **Breast exam** | Breasts? |  |  |
|  | **Uterus exam** | Uterus? |  |  |
|  | **Lochia exam** | Vaginal discharge? |  |  |
|  | **Perineum exam** | Perineum? |  |  |
|  | **Family planning** | Did any health provider discuss contraceptive method and contraceptive use with you? |  |  |
| **Child immunization** | |  |  |  |
|  | **BCG vaccine** | Please tell me if [NAME] had any of the following vaccinations:  A BCG vaccine injection against tuberculosis? | Please tell me if [NAME] had any of the following vaccinations:  A BCG vaccination against tuberculosis, that is, an injection in the arm or shoulder that usually causes a scar? | Has [NAME] ever been given a bcg vaccination against tuberculosis – that is, an injection in the arm or shoulder that caused a scar? |
|  | **Polio vaccine** | Polio vaccine, that is, a candy ball formed vaccine for the child to eat? | Polio vaccine, that is, drops in the mouth? | Has [NAME] ever been given any “vaccination drops in the mouth” to protect him/her from getting diseases – that is, polio? |
|  | **HBV vaccine** | A Hepatitis B vaccine injection? |  |  |
|  | **DPT vaccine** | A DPT vaccine injection? | A DPT vaccination, that is, an injection given in the thigh or buttocks, sometimes at the same time as polio drops? | Has [NAME] ever been given “dpt vaccination injections” – that is, an injection in the thigh or buttocks – to prevent him/her from getting tetanus, whooping cough, diphtheria? (sometimes given at the same time as polio) |
|  | **Measles vaccine** | A measles vaccine injection? | A measles injection or an MMR injection - that is, a shot in the arm at the age of 9 months or older - to prevent him/her from getting measles? | Has [NAME] ever been given “measles vaccination injections” or MMR – that is, a shot in the arm at the age of 9 months or older - to prevent him/her from getting measles? |
